# Effect of a Low-Glycemic Index Nutritional Intervention on Body Weight and Selected Cardiometabolic Parameters in Children and Adolescents with Excess Body Weight and Dyslipidemia

**DOI:** 10.3390/nu16132127

**Published:** 2024-07-03

**Authors:** Beata Bondyra-Wiśniewska, Anna Harton

**Affiliations:** Department of Dietetics, Institute of Human Nutrition Sciences, Warsaw University of Life Sciences (WULS), 159C Nowoursynowska St, 02-776 Warsaw, Poland

**Keywords:** glycemic index, lipids, dyslipidemias, child, adolescent, obesity, body mass index, diet, diet therapy, nutritional intervention

## Abstract

Excess body weight in pediatric patients and the resulting dyslipidemia, if left untreated, are a serious risk factor for cardiovascular disease in young adults. Despite this, there is still no effective and validated nutritional strategy for the treatment of overweight/obesity and comorbid dyslipidemia in children and adolescents. A low-glycemic index (LGI) diet may be recommended, but evidence for its effectiveness in the pediatric population is limited. The aim of this study was to evaluate the effectiveness of nutritional intervention in children and adolescents with excess body weight and dyslipidemia. The study was conducted in patients aged 8–16 with overweight or obesity and lipid disorders (*n* = 64), of which 40 participants who completed the entire 8-week study were included in the analysis. Patients were randomly selected and allocated to one of the two dietary groups: the LGI diet or the standard therapy (ST) diet. Both diets were based on the principal recommendation of Cardiovascular Health Integrated Lifestyle Diet-2 (CHILD-2). This study showed that both LGI and ST diets were equally beneficial in reducing body weight, body fat, blood pressure, total cholesterol (TC), and triglyceride (TG) levels. The LGI diet, compared to the ST diet, was less effective in reducing blood TG levels but more effective in reducing diastolic blood pressure (DBP). Therefore, the choice of the type of diet in the treatment of children and adolescents with excess body weight and dyslipidemia may be individual. However, it should be based on the recommendation of CHILD-2. Further long-term, larger-scale studies are needed.

## 1. Introduction

Excess body weight in children and adolescents has been a serious global problem for decades [1,2]. Analysis of body weight and height among 63 million children and adolescents aged 5–19 from around the world indicates that the obesity rate in 2022 was more than four times higher than in 1990, both in girls (from 1 7% to 6.9%) and boys (from 2.1% to 9.3%). An upward trend was observed in almost all of the 200 countries assessed. The total number of children and adolescents with obesity in 2022 is almost 159.3 million (65.1 million girls and 94.2 million boys) compared to 31.4 million in 1990. What is also disturbing is the fact that the obesity epidemic, which mainly affected adults about 30 years ago, is now visible among school-age children and adolescents [3]. Dyslipidemia and high blood pressure, which are important risk factors for cardiovascular diseases, are just some of the health consequences of overweight and obesity [4,5]. It is known that if left untreated, they can lead to serious diseases in young adults [6,7]. Failure to implement effective weight loss methods at an early stage may result in maintaining excessive body weight in adulthood. This creates a serious risk of developing many chronic diseases, including type 2 diabetes, hypertension, metabolic syndrome, cancer, and many others in increasingly younger age groups. This, in turn, contributes to a deterioration in the quality of life and a reduction in life expectancy [8,9]. The lack of effective and validated nutritional strategies for the treatment of excess body weight and comorbid lipid disorders in the pediatric population, as well as the lack of research in such a group, is disturbing [10]. Until recently, it was thought that a low-glycemic index (LGI) diet was a better form of diet therapy for excess body weight than a higher glycemic index (GI) diet [11]. Recent reports cast doubt on these conclusions, although small sample sizes and only a few participants in the comparison groups make these data moderate to very low certainty [12]. Due to limited evidence regarding the impact of the GI on body weight and cardiometabolic parameters in the pediatric population, there is a great need to conduct further, well-planned research in this area among patients from different countries and cultures and with different eating habits. Most interventions are based on standard dietary recommendations, which is a routine approach to the treatment of obesity also in children [10]. However, the search for alternative, effective methods of diet therapy for excessive body weight in pediatric patients is still ongoing. Therefore, this study evaluated the effectiveness of an energy-balanced diet based on the principal recommendation on Cardiovascular Health Integrated Lifestyle Diet-2 (CHILD-2) and additionally on LGI products in children and adolescents with excess body weight and dyslipidemia.

## 2. Methods

The study was conducted in 2019–2020 among 64 patients aged 8–16 diagnosed with overweight or obesity and dyslipidemia. It was completed by 40 participants (62.5% participant retention rate), including 24 boys and 16 girls, who were included in the analysis. The PICO (population, intervention, comparison, outcomes) criteria used to define the research question are presented in Table 1.

The primary aim of this study was to evaluate the impact of nutritional intervention on body weight reduction and improvement of cardiometabolic parameters. The secondary aims of the study were to assess the intervention’s effect on body composition and waist and hip circumference. Overweight was diagnosed with a body mass index (BMI) of 85–95. percentile, and obesity > 95. percentile for age and gender based on the Polish growth reference values defined by the International Obesity Task Force (IOTF) [14,15]. Dyslipidemia was defined as the presence of at least one lipid abnormality, such as high total cholesterol (TC), high low-density lipoprotein cholesterol (LDL-C), high triglyceride (TG), or low high-density lipoprotein cholesterol (HDL-C) according to the American College of Cardiology [16]. Patients were under the care of a pediatrician from the Children’s Memorial Health Institute in Warsaw (one of the largest pediatric hospitals in Poland) and a dietitian with higher education (after 5 years of Master’s studies) and 5 years of experience working with pediatric patients. Patients were qualified to participate in this study by a pediatrician based on a medical interview.

Patients were randomly selected and allocated to one of the two groups: the intervention group with a low-GI (LGI) diet or the control group with a standard therapy (ST) diet. In this study, double blinding was not possible because the dietitian conducting the intervention explained to the patient how to follow the diet and answered the patient’s questions about the diet. The patients did not know which of the two diets they were following.

Both diets were based on the principal recommendation of the Cardiovascular Health Integrated Lifestyle Diet-2 (CHILD-2) [13]. An additional assumption of the LGI diet was the consumption of products with a low GI (<55), such as whole-grain products, low-starch vegetables, and raw fruit with a reduced content of sugars, nuts, and seeds [13,17,18]. Table 2 shows the differences in products and their preparation in both diets affecting the GI. Table 2 includes products that were a significant source of simple sugars in dietary plans. The GI values of the foods were obtained from international tables [17]. On this basis, the glycemic load (GL) was calculated, which additionally considers the amount of consumed product. The formula was used: GL = available carbohydrates [g] × GI/100. The GL of daily food ration was calculated by summing the glycemic loads for all foods consumed in the diet. It was assumed that the GL of the daily food ration was <120 for the LGI diet and ≥120 for the ST diet [19,20].

The energy value of the diets was individually adjusted to the degree of excess body weight based on the basal metabolic rate and level of physical activity [21,22]. The diets were matched for macronutrient composition: 15–20% from protein, 25–30% from fats (saturated fatty acids <7%), and 50–60% from carbohydrates for daily energy. Each patient received a diet plan that assumed the consumption of five meals a day and included 10 dishes for each meal with similar energy and nutritional value. Patients were free to choose their preferred dish at each meal. Over 8 weeks, participants attended three visits with a dietitian, during which their adherence to the diet and current and habitual food intake was assessed, and anthropometric measurements (i.e., height, weight, waist, hip and arm circumference, body composition) and blood pressure were taken. Throughout the entire study period, patients were required to keep food diaries in which they recorded all meals and drinks consumed. During follow-up visits, the dietitian analyzed these records and assessed the main aspects of the dietary recommendations. Such an assessment, with particular emphasis on the most important assumptions of the diet, constituted a specific control over the patients’ compliance with dietary recommendations. An analysis of the nutritional value and GI of the diet will be presented in the subsequent article. Blood lipid parameters (TC, LDL-C, HDL-C, and TG) were ordered by a pediatrician who interpreted the results and made a diagnosis of a lipid disorder. All visits with participants took place in the presence of a parent/primary caregiver. More details about the design and study are provided in the study protocol [23].

The study protocol was approved by the Ethics Committee of the Faculty of Human Nutrition and Consumer Science, Warsaw University of Life Sciences WULS, Poland (10p/2017, 17 May 2017). Each patient and the parent/primary caregiver were informed about the study’s aim and procedure, and written consent was obtained. The patient could withdraw from further participation in the study at any stage without giving a reason.

## 3. Statistical Analysis

All statistical analyses were conducted using Statistica version 13.1 (Copyright©StatSoft, Inc., 1984–2014, Cracow, Poland). For all tests, *p* < 0.05 was considered as significant.

Nonparametric tests were used in statistical analyses due to the lack of normal distribution in groups. The Mann–Whitney U test was used to compare the differences in the baseline characteristics of the study group and changes in parameters between LGI and ST diet groups after the end of the nutritional intervention. The Wilcoxon matched-pairs test was used to compare the values of anthropometric and cardiometabolic parameters before (baseline) and after 8 weeks of the nutritional intervention program in the LGI and ST diet groups. All quantitative data are expressed as mean ± standard deviation (SD). The chi-squared test was used to assess differences between qualitative variables. Statistical tests used for analysis are described separately in each data table. In all statistical analyses, gender was also considered, but it had no impact on the anthropometric and cardiometabolic data (*p* > 0.05). Due to the lack of differences, these data were not included in the paper.

## 4. Results

### 4.1. Baseline Characteristics

The baseline characteristics of the study group that completed the nutritional intervention program are presented in Table 3. Before the start of the nutritional intervention, there were no significant differences between the LGI and ST diet groups in all parameters except waist-to-hip ratio (WHR; this was significantly higher in the LGI diet group).

The baseline characteristics of the cardiometabolic parameters for participants who completed the nutritional intervention program are presented in Table 4. There were no significant differences between the LGI and ST diet groups in mean values for cardiometabolic parameters, except diastolic blood pressure (DBP) and DBP-for-age percentile, which were higher in the LGI diet group.

### 4.2. Changes in Anthropometric Parameters

After 8 weeks, in both groups, there was a significant decrease in weight and BMI (Table 5). Similarly, after both LGI and ST diets, there was a significant reduction in the weight-for-age percentile and BMI-for-age percentile. Waist circumference decreased significantly in LGI and ST diet groups, as did hip circumference. However, the WHR did not show significant changes in both groups. The waist-to-height ratio (WHtR) was also significantly reduced in both groups, which could additionally result from a significant increase in height during the study. There was no significant change in arm circumference in either group.

Body composition measurements using bioelectrical impedance analysis (BIA) showed that both body fat mass (FM) and its percentage were significantly reduced in the LGI and ST diet groups. At the same time, the percentage of fat-free mass (FFM), total body water (TBW), muscle mass (MM), and skeletal muscle mass (SMM) increased significantly in both groups. However, FFM expressed in kilograms was significantly reduced only in the ST diet group, similar to TBW, MM, and SMM (Table 5).

### 4.3. Changes in Cardiometabolic Parameters

At the end of the nutritional intervention, both groups significantly decreased systolic (SBP) and diastolic blood pressure (Table 6). Similarly, for blood pressure percentiles, there was a reduction in the SBP-for-age percentile and DBP-for-age percentile after both LGI and ST diets. There was no significant change in heart rate in either group.

A comparison of lipid parameters within groups showed a significant reduction in TC and TG levels between the first and third visits in both groups. No significant change in LDL-C levels was observed in any of the groups; however, HDL-C concentration decreased significantly in the LGI diet group (Table 6). 

### 4.4. Comparing the Effects of the LGI and ST Diets

Table 7 shows changes in variables between groups, which represent differences between data after 8 weeks of nutritional intervention and at baseline. Among the anthropometric parameters, a significantly greater decrease was observed in the ST diet group in the weight-for-age percentile and BMI-for-age percentile. Also, in the ST diet group, decrements in TG concentrations were significantly greater. However, in DBP and HDL-C, the decrease was significantly greater in the LGI diet group. For the other parameters, there were no statistically significant differences in changes between the baseline and 8-week LGI and ST diet groups.

The individual effects achieved by patients during the nutritional intervention were analyzed. Half of the participants achieved a weight loss of at least 5% from their baseline body weight (14 from the LGI diet group (64%) and six from the ST diet group (33%)), although these differences were on the verge of statistical significance (*p* = 0.057; chi-squared test).

Analyzing individual cases of children and adolescents participating in the study, some significant differences in the distribution of body weight were observed in the entire group, regardless of the type of diet used. During 8 weeks of intervention, it was possible to significantly reduce the percentage of children and adolescents with obesity and increase the percentage of participants with normal body weight. Changes in body weight and selected cardiometabolic parameters in the total group and the LGI and ST diet groups are presented in Table 8. In particular, the overall percentage of children and adolescents with high TG levels decreased.

## 5. Discussion

In this study, two types of diets for children and adolescents with excess body weight and dyslipidemia were compared—one the low-GI (LGI) diet (intervention group) and one standard therapy (ST) diet (control group). Both diets are based on the principal recommendations of CHILD-2 [13]. The results are discussed in the sections on anthropometric parameters, body composition, and cardiometabolic parameters.

### 5.1. The Impact of the Assessed Diets on Anthropometric Parameters

Both of the examined diets were effective in reducing body weight and BMI after 8 weeks of nutritional intervention—there were no significant differences (in these parameters) between the LGI and ST diet groups. Both diets also had a positive impact on reducing the incidence of obesity in children and adolescents.

In the available literature, the effect of the LGI diet on weight loss in children and adolescents is not clear. Most studies using LGI diets are conducted among adults, and here too, the results are divergent. Some of these studies showed a significant decrease in weight or BMI after the LGI diet [24,25,26,27,28] or low-glycemic load (LGL) diet [29,30], while others showed no change [31,32]. Comparing the effectiveness of the LGI diet to a diet with a higher GI (HGI) also does not give clear conclusions. In some studies, greater effectiveness in reducing body weight or BMI was observed after the LGI diet compared to the HGI diet [33,34]. In a 2007 Cochrane review, the authors concluded that people with overweight or obesity on the LGI diets lost more weight than those on the HGI diets [11]. A recent 2023 update to this review concluded that there is no convincing evidence that the LGI diets are more effective than the HGI or other diets [12]. However, the authors rated the certainty of this evidence as moderate to very low. Meanwhile, among adolescents with obesity and insulin resistance, a higher GL of the diet was associated with less weight loss [35]. It has also been noted that there is an association between excessive weight gain and changes or levels of consumption of certain food and drink groups among children aged 7–13 years [36]. The food products that were most positively associated with three-year excess weight gain included desserts, sweets, and sugar-sweetened drinks, i.e., products with a high GI. In turn, the foods that caused weight loss were whole grains and cereals rich in fiber, i.e., those with a lower GI. Several studies have also evaluated the effect of the LGI or LGL diets compared to reduced fat diets in the treatment of childhood obesity. Both diets seem to be effective in reducing body weight or BMI; however, no significant differences were observed between the types of nutritional interventions [25,27,29,30]. Compared to a standard diet based on healthy nutritional recommendations, the LGI diet was also no longer effective [28,31]. Other researchers also came to a similar conclusion after conducting a meta-analysis of 85 studies, which showed that LGI diets were effective in reducing body weight and BMI, however, the effect was not greater compared to various control diets [24].

Both diets studied (LGI and ST) were effective in reducing waist and hip circumference and WHtR, but neither showed a significant reduction in WHR. The LGI diet was not observed to be more effective in changing these parameters than the ST diet. Several previous publications also showed the effect of the LGI or LGL diet on reducing waist circumference [25,29,34], while in other studies, no significant changes were observed [27,32]. Similar to our results, also in the case of WHR, no significant changes were observed after the LGI diet [29,32]. The WHtR value also did not change significantly [32], which was a different observation compared to our results. It could also be caused by a significant increase in height. Most studies found no differences in effect sizes between the LGI diet and control groups in the above parameters. Only in adolescents aged 15–18 with obesity was the LGI diet more effective than the HGI diet in reducing waist circumference after adjusting for age and gender [33]. It is worth emphasizing that waist circumference is the main diagnostic criterion for metabolic syndrome in children and adolescents defined by the International Diabetes Federation [37]. It is also known that there is a correlation between waist circumference and visceral fat tissue. This, in turn, contributes to the development of other disorders classified as metabolic syndrome (including lipid disorders, high blood pressure, and insulin resistance) [37]. The positive effect of both diets used in this study on changes in waist circumference provides the prospect of reducing the risk of developing other disorders that are a component of metabolic syndrome or improving those that already occur. However, further efforts are needed to reduce waist circumference.

The available literature suggests that losing at least 5% of initial body weight in adults with obesity reduces the risk or progression of weight-related complications [38,39,40]. This value is considered clinically significant. Unfortunately, less is known about clinically significant weight loss in children and adolescents with excess body weight. However, in our study, we observed that half of the participants achieved a weight loss of at least 5% after 8 weeks of the nutritional intervention compared to baseline. There was also a trend (statistically insignificant results, *p* > 0.05) for twice as many participants on the LGI diet to achieve at least a 5% reduction in body weight from baseline compared to the ST diet group.

### 5.2. The Impact of the Assessed Diets on Body Composition

Our study showed a positive effect of both diets (LGI and ST) on body composition in study groups. Both diets were effective in reducing body FM, resulting in a significant increase in total body water percentage. However, the LGI diet was not shown to be more effective than the ST diet in reducing FM from baseline to week 8 of the intervention. In another study, a significant reduction in the percentage of FM was observed in children with obesity following the LGI diet after 3 and 6 months [32]. However, unlike our study, the diet was additionally reduced in fructose, and the participants had nonalcoholic fatty liver disease (NAFLD). A previous randomized controlled trial in adolescents aged 15–18 with obesity found no significant difference in body fat percentage over 6 months on the LGI diet [33]. Other researchers also indicate that the LGI diet has no effect on the reduction of FM in children and adolescents with obesity [25,27]. The greater effectiveness of the LGI diet compared to the low-fat diet [25,27] and other control diets [24,33] has not been confirmed.

Weight loss is associated with changes in body composition. Overall, there is reasonable concern that weight loss will reduce satiety and basal metabolic rate (BMR), also due to adaptive hormonal changes [41]. This, in turn, may promote weight regain. BMR can be further slowed by the loss of FFM (including muscles), considered the best determinant of BMR [42,43,44]. In our study, no significant differences in FFM, MM, and SMM (expressed in kilograms) were observed in participants on the LGI diet, while the ST diet significantly reduced these parameters. However, when analyzing the change in the percentages of FFM, MM, and SMM, a significant increase was observed in both groups. Unfortunately, most studies examining the effectiveness of the LGI or LGL diet for weight loss in children and adolescents have not assessed changes in FFM or MM [24,25,26,28,29,30,31,33,34]. However, in 91 adults with obesity, the GI of the diet was shown to have no effect on differences in body composition—both in the LGI and HGI diets, the proportion of FM to FFM losses was the same [45]. FFM, including MM, appears to depend more on energy deficit and protein intake than on the GI [46]. A systematic review and meta-analysis of randomized trials showed that an energy-reduced diet with a protein content of 20–30% for daily energy does not result in a loss of FFM over 4 months in children aged 6–18 years [47]. In another review of studies, researchers concluded that weight loss in patients with obesity may result in decreased MM, but without negative effects on muscle strength and overall physical performance, possibly due to the reduction in FM. In turn, higher protein intake helped preserve MM during weight loss [48]. It appears that a balanced diet and personalized nutritional education contributed to a positive effect on body composition in the participants of this study, including physiological growth. Therefore, it may be expected that they will not regain the weight later.

### 5.3. The Impact of the Assessed Diets on Cardiometabolic Parameters

This study demonstrated that in children and adolescents with excess body weight and dyslipidemia, the nutritional intervention positively affected some cardiometabolic parameters. Significant reductions in SBP, SBP-for-age percentile, DBP, and DBP-for-age percentile were observed over 8 weeks in the LGI and ST diet groups. However, the greater effectiveness of the LGI diet was observed only for DBP. Of note, baseline DBP was significantly higher in the LGI diet group, which may be a confounding factor affecting the results. In the available literature [25,27,29,32,33,34], the LGI diet’s effect on blood pressure varies. As in our study, several others showed a positive impact of the LGI diet on SBP [32,34] and DBP [25,29,34]. Some, however, found no significant changes in SBP [25,27,29,33] and DBP [27,33]. Also, the LGI diet was not shown to be more effective than other diets in reducing blood pressure in the pediatric population with excess body weight [25,29]. However, in children and adolescents with obesity and NAFLD, low glycemic load of the diet was related to reductions in SBP [32]. Excess body weight is an established risk factor for high blood pressure in children and adults [49]. For this reason, the primary approach to lowering blood pressure should focus on achieving a healthy body weight [50]. A previous study showed a relationship between a reduction in BMI and a decrease in SBP and DBP after a year of lifestyle intervention in children and adolescents with excess body weight [41]. A significant reduction in body weight and blood pressure was observed in the participants of this study. Therefore, continued efforts to achieve a healthy weight can be expected to provide additional benefits, including reducing the risk of cardiovascular disease in adulthood [49].

Our study demonstrated a significant reduction in the TC and no effect on LDL-C in the LGI and ST diet groups. TG concentration decreased in both groups, but this decrease in the ST diet group was significantly greater. Notably, baseline TG concentration was higher in the LGI diet group than in the ST diet group (the difference was not statistically significant). Both diets also positively impacted reducing the number of children and adolescents with high TG levels. Most available studies on the effectiveness of the LGI diet among children and adolescents with excess body weight have shown that the LGI diet does not induce significant changes in TG levels [25,26,27,28,29,32,33,34], TC [25,27,28,32,33,34], LDL-C [25,26,27,28,29,32,33] and HDL-C [25,27,28,29,32,33,34]. Researchers reached different conclusions in a meta-analysis of 85 studies, including children, adolescents, and adults with obesity, who showed that LGI diets effectively reduce TG, TC, and LDL-C levels [24]. A significant reduction in TC concentration also occurred in children and adolescents aged 8–14 with obesity after one year of the LGI diet [26]. Our study showed that the LGI diet had an unfavorable effect on reducing HDL-C concentration, while it did not change significantly in the ST diet group. In turn, Zafar et al. [24] showed that the LGI diet did not substantially affect HDL-C levels. Several previous studies have not observed that the LGI diet is more effective in improving lipid parameters compared to the low-fat diet [25,27,29], energy-restricted diet [26], or other recommendations related to a healthy diet [28]. A significant difference was observed only among 22 children with obesity after adjusting for age and gender. There was a greater difference in TG concentration from baseline values after the LGI diet compared to the HGI diet [34]. In turn, a meta-analysis of studies showed that the effect of the LGI diet compared to the HGI diet was significantly greater for LDL-C and TC, and compared to diets used to treat hypertension, the LGI diet was more effective in the case of TC and TG [24]. However, the difference between these studies and ours was that the initial values of lipid parameters were not assessed in the participants. In our study, the inclusion criterion was the presence of at least one lipid abnormality. An additional issue is that in most of the available studies, their authors compare the effectiveness of LGI and HGI diets. Our study compares the LGI diet with the ST diet, which ultimately does not result in such spectacular differences.

### 5.4. Strengths and Limitations

The presented study has several strengths: firstly, the type of study. There is still insufficient data in the literature assessing the impact of the LGI diet on weight loss and improvement of cardiometabolic parameters among pediatric patients with excess body weight and dyslipidemia. Our research is a response to this situation. Secondly, the study assessed the effectiveness of the nutritional intervention using two types of diets (LGI diet—intervention group [18,27,34] and ST diet—control group), both based on the principal recommendations on CHILD-2 [13]. Moreover, many studies on the dietary treatment of excess body weight only concern children and adolescents with obesity. The occurrence of lipid disorders is also not considered. Our program included participants with overweight or obesity and an additional criterion was the presence of at least one lipid abnormality diagnosed by a pediatrician. In the treatment of obesity, it is worth focusing not only on weight loss but also on additional disorders that often coexist with it. Dyslipidemia is one of the health consequences of excess body weight and increases the risk of cardiometabolic diseases [51,52]. In our nutritional intervention program, we planned and implemented a comprehensive approach to patient care, including the care of a pediatrician and a dietitian. Also, the support of the parent/primary caregiver involved in nutritional counseling and childcare during the study may increase the effectiveness of the nutritional intervention in children and adolescents. The presented research concerned minors; therefore, the involvement of parents/primary caregivers was necessary at every stage.

On the other hand, parental involvement may be a limiting factor, especially if it is low. Less parental support and involvement may result in dropouts, which was the case for several participants in this study. Another limiting factor is the different ages of the respondents. Age may differentiate the broadly understood lifestyle, including patients’ approach to their appearance and health, as well as influence their involvement and, therefore, the intervention results. The timing of the study may also affect the results of the intervention. Our study was 8 weeks, which could have resulted in limited improvement in anthropometric parameters and all cardiometabolic indices. A review of thematic literature [53] indicated that long-term therapies (>6 months) compared to shorter ones give better results. Additionally, the sustainability of changes in anthropometric and cardiometabolic parameters in the long term is unknown. Further follow-up studies should be conducted to confirm whether these beneficial effects will be maintained in the long term. Nonetheless, our results support the promise of improving the health of children and adolescents with overweight or obesity within 8 weeks of following the LGI or ST diet. Finally, the BIA method was used to assess body composition, which is considered less accurate compared to methods such as DEXA devices. However, the BIA method is also recognized and often used in research due to the lower cost of the procedure. Additionally, measurements were performed at three time points on the same device to minimize the risk of measurement bias.

## 6. Conclusions

This study showed that LGI and ST diets were equally beneficial for children and adolescents with excess body weight and dyslipidemia in reductions in body weight, body fat, blood pressure as well as TC and TG levels within 8 weeks. Although the LGI diet, compared to the ST diet, was less effective in reducing blood TG levels but more effective in lowering DBP. It is worth noting that the subjects on the LGI diet were characterized by significantly higher values of DBP at the beginning of the study, which could have influenced the final results. Due to the similar impact on body weight, anthropometric and cardiometabolic parameters of the LGI diet and the ST diet, the choice of diet in the diet therapy of children and adolescents with excessive body weight and dyslipidemia may be individual. However, it should be based on the recommendation of Cardiovascular Health Integrated Lifestyle Diet-2 (CHILD-2), the main goal of which is to prevent risk factors for cardiovascular disease. Long-term, large-scale studies are needed to evaluate the effectiveness of the LGI diet in reducing body weight and improving lipid parameters in children and adolescents with overweight/obesity and dyslipidemia.

## Figures and Tables

**Table 1:** Study characteristics according to PICO criteria.

Criterion	Implementation to Define the Research Question
Population	Children and adolescents aged 7–18 with overweight or obesity and lipid disorders.
Intervention	Nutritional intervention (8 weeks) and comparison of the effectiveness of two types of diets.
Comparison	Study groups: low-GI (LGI) diet * (intervention group) and standard therapy (ST) diet * (control group).
Outcomes	Effectiveness of nutritional intervention in reducing body weight and improving cardiometabolic parameters.

* Both diets were based on the principal recommendation of the Cardiovascular Health Integrated Lifestyle Diet-2 (CHILD-2) [13].

**Table 2:** Product differences in GI.

Products	LGI Diet	ST Diet
Fruits	-With less sugar (e.g., blueberries, raspberries) -Slightly unripe -Raw	-With more sugar (e.g., banana, grapes) -Ripe -Baked, boiled
Grains	-Whole grain products, e.g., buckwheat, barley, pearl barley, whole meal pasta, brown rice, whole wheat bread and graham, oatmeal -Cooked al dente	-Not whole grain products, e.g., millet, semolina, pearl barley groats, white-flour pasta, white rice, white bread, cornflakes -Boiled “soft”
Potatoes	-Baked or boiled whole	-Boiled and mashed
Starchy vegetables (e.g., carrots, beets)	-Raw	-Boiled

**Table 3:** Baseline characteristics of the study group that completed the nutritional intervention program (mean ± SD).

Variable	Total (*n* = 40)	LGI Diet (*n* = 22)	ST Diet (*n* = 18)	*p*-Value (Mann–Whitney U Test)
Age [years]	13.35 ± 2.63	13.18 ± 2.71	13.56 ± 2.57	ns
Birth weight [g]	3413.00 ± 412.80	3493.63 ± 425.59	3314.44 ± 385.32	ns
Moderate or high-intensity physical activity [min/day]	45.44 ± 44.91	45.80 ± 46.51	45.00 ± 44.22	ns
Screen time [min/day]	184.50 ± 68.80	174.50 ± 62.65	196.67 ± 75.87	ns
BMR [kcal]	1827.60 ± 388.80	1916.36 ± 419.51	1719.11 ± 326.71	ns
Anthropometrics				
Height (cm)	165.98 ± 15.50	166.86 ± 15.62	164.90 ± 15.73	ns
Body weight (kg)	78.08 ± 26.36	82.32 ± 30.67	72.89 ± 19.49	ns
Body weight-for-age percentile	93.88 ± 6.85	94.43 ± 6.97	93.21 ± 6.84	ns
BMI (kg/m^2^)	27.48 ± 5.67	28.43 ± 6.70	26.31 ± 3.96	ns
BMI-for-age percentile	94.29 ± 4.90	94.71 ± 5.31	93.77 ± 4.45	ns
Arm circumference (cm)	29.80 ± 4.64	30.03 ± 4.91	29.51 ± 4.40	ns
Waist circumference (cm)	93.50 ± 17.55	96.42 ± 18.74	89.93 ± 15.76	ns
Hip circumference (cm)	101.96 ± 13.04	101.27 ± 14.61	102.80 ± 11.18	ns
WHtR	0.56 ± 0.07	0.57 ± 0.07	0.54 ± 0.07	ns
WHR	0.91 ± 0.09	0.95 ± 0.08	0.87 ± 0.09	0.020
FM (kg)	27.12 ± 12.75	29.15 ± 15.59	24.63 ± 7.79	ns
FM (%)	33.60 ± 5.17	33.71 ± 6.02	33.47 ± 4.06	ns
FFM (kg)	50.96 ± 14.90	53.16 ± 16.52	48.27 ± 12.58	ns
FFM (%)	66.38 ± 5.15	66.25 ± 6.00	66.54 ± 4.04	ns
TBW (kg)	37.32 ± 10.92	38.95 ± 12.11	35.32 ± 9.21	ns
TBW (%)	48.60 ± 3.79	48.53 ± 4.41	48.68 ± 2.98	ns
MM (kg)	48.36 ± 14.23	50.47 ± 15.79	45.77 ± 11.98	ns
MM (%)	62.94 ± 4.82	62.83 ± 5.60	63.07 ± 3.80	ns
SMM (kg)	28.80 ± 8.44	30.08 ± 9.38	27.22 ± 7.08	ns
SMM (%)	37.65 ± 2.89	37.55 ± 3.36	37.78 ± 2.26	ns

BMR—basal metabolic rate; BMI—body mass index; WHtR—waist-to-height ratio; WHR—waist-to-hip ratio; FM—fat mass; FFM—fat-free mass; TBW—total body water; MM—muscle mass; SMM—skeletal muscle mass; ns—not significant (*p* ≥ 0.05); LGI diet—low-glycemic index diet; ST diet—standard therapy diet.

**Table 4:** Baseline characteristics of cardiometabolic parameters of participants who completed the nutritional intervention program (mean ± SD).

Variable	Total (*n* = 40)	LGI Diet (*n* = 22)	ST Diet (*n* = 18)	*p*-Value (Mann–Whitney U Test)
SBP (mmHg)	117.75 ± 7.97	117.82 ± 4.57	117.67 ± 10.94	ns
SBP-for-age percentile	66.55 ± 23.50	66.36 ± 19.77	66.78 ± 28.01	ns
DBP (mmHg)	70.90 ± 6.02	73.55 ± 4.19	67.67 ± 6.42	0.013
DBP-for-age percentile	78.15 ± 21.89	87.09 ± 9.69	67.22 ± 27.46	0.006
Heart rate (bpm)	72.90 ± 4.82	73.27 ± 4.70	72.44 ± 5.07	ns
TC (mg/dl)	190.59 ± 23.09	190.63 ± 19.31	190.54 ± 27.61	ns
HDL-C (mg/dl)	40.02 ± 9.21	39.24 ± 10.67	40.98 ± 7.23	ns
LDL-C (mg/dl)	110.19 ± 14.63	110.51 ± 13.62	109.79 ± 16.17	ns
TG (mg/dl)	204.26 ± 58.70	216.64 ± 65.41	206.19 ± 56.00	ns

SBP—systolic blood pressure; DBP—diastolic blood pressure; TC—total cholesterol; HDL-C—high-density lipoprotein cholesterol; LDL-C—low-density lipoprotein cholesterol; TG—triglyceride; ns—not significant (*p* ≥ 0.05); LGI diet—low-glycemic index diet; ST diet—standard therapy diet.

**Table 5:** Changes in anthropometric parameters after 4 and 8 weeks of nutritional intervention (mean ± SD).

Variable	LGI Diet (*n* = 22)				ST Diet (*n* = 18)			
	Baseline	After 4 Weeks	After 8 Weeks	*p*-Value * (Wilcoxon Test)	Baseline	After 4 Weeks	After 8 Weeks	*p*-Value * (Wilcoxon Test)
Height (cm)	166.86 ± 15.62	167.25 ± 15.40	167.34 ± 15.38	0.002	164.90 ± 15.73	165.34 ± 15.98	165.48 ± 15.97	<0.001
Weight (kg)	82.32 ± 30.67	79.70 ± 28.70	78.11 ± 27.46	<0.001	72.89 ± 19.49	71.22 ± 18.69	69.80 ± 18.42	<0.001
Weight-for-age percentile	94.43 ± 6.97	93.53 ± 7.02	92.98 ± 7.18	0.003	93.21 ± 6.84	91.77 ± 8.39	89.78 ± 9.60	<0.001
BMI (kg/m^2^)	28.43 ± 6.70	27.47 ± 6.21	26.95 ± 5.83	<0.001	26.31 ± 3.96	25.59 ± 3.74	25.06 ± 3.74	<0.001
BMI-for-age percentile	94.71 ± 5.31	93.07 ± 7.81	92.07 ± 9.43	0.005	93.77 ± 4.45	92.21 ± 5.59	89.99 ± 7.39	<0.001
Arm circumference (cm)	30.03 ± 4.91	30.99 ± 5.50	30.37 ± 5.35	ns	29.51 ± 4.40	29.68 ± 4.17	29.22 ± 4.04	ns
Waist circumference (cm)	96.42 ± 18.74	94.99 ± 16.57	91.22 ± 15.69	0.026	89.93 ± 15.76	88.47 ± 15.50	86.29 ± 15.60	<0.001
Hip circumference (cm)	101.27 ± 14.61	101.85 ± 15.17	99.16 ± 14.63	0.031	102.80 ± 11.18	100.97 ± 10.60	99.01 ± 10.50	<0.001
WHtR	0.57 ± 0.07	0.57 ± 0.07	0.54 ± 0.07	0.026	0.54 ± 0.07	0.53 ± 0.07	0.52 ± 0.07	<0.001
WHR	0.95 ± 0.08	0.93 ± 0.06	0.92 ± 0.07	ns	0.87 ± 0.09	0.87 ± 0.08	0.87 ± 0.08	ns
FM (kg)	29.15 ± 15.59	26.61 ± 13.49	25.95 ± 12.11	<0.001	24.63 ± 7.79	23.34 ± 7.33	22.77 ± 7.34	<0.001
FM (%)	33.71 ± 6.02	32.05 ± 5.12	32.12 ± 4.43	0.031	33.47 ± 4.06	32.41 ± 4.51	32.24 ± 4.61	<0.001
FFM (kg)	53.16 ± 16.52	53.10 ± 16.34	52.16 ± 15.97	ns	48.27 ± 12.58	47.89 ± 12.43	47.03 ± 12.12	<0.001
FFM (%)	66.25 ± 6.00	67.95 ± 5.14	67.88 ± 4.39	0.031	66.54 ± 4.04	67.59 ± 4.47	67.76 ± 4.64	<0.001
TBW (kg)	38.95 ± 12.11	38.99 ± 11.96	38.34 ± 11.66	ns	35.32 ± 9.21	35.34 ± 9.27	34.79 ± 9.07	<0.001
TBW (%)	48.53 ± 4.41	49.90 ± 3.81	49.91 ± 3.28	0.011	48.68 ± 2.98	49.83 ± 3.26	50.06 ± 3.42	<0.001
MM (kg)	50.47 ± 15.79	50.55 ± 15.60	49.74 ± 15.22	ns	45.77 ± 11.98	45.86 ± 12.08	45.11 ± 11.83	<0.001
MM (%)	62.83 ± 5.60	64.64 ± 5.60	64.69 ± 4.22	0.007	63.07 ± 3.80	64.63 ± 4.22	64.89 ± 4.31	<0.001
SMM (kg)	30.08 ± 9.38	30.15 ± 9.26	29.65 ± 9.04	ns	27.22 ± 7.08	27.28 ± 7.14	26.84 ± 6.99	<0.001
SMM (%)	37.55 ± 3.36	38.60 ± 2.89	38.51 ± 2.57	0.010	37.78 ± 2.26	38.48 ± 2.50	38.62 ± 2.60	0.002

BMI—body mass index; WHtR—waist-to-height ratio; WHR—waist-to-hip ratio; FM—fat mass; FFM—fat-free mass; TBW—total body water; MM—muscle mass; SMM—skeletal muscle mass; ns—not significant (*p* ≥ 0.05); LGI diet—low-glycemic Index diet; ST diet—standard therapy diet; * significant differences between baseline and after 8 weeks from the LGI diet and ST diet groups, respectively.

**Table 6:** Changes in cardiometabolic parameters after 4 and 8 weeks of nutritional intervention (mean ± SD).

Variable	LGI Diet (*n* = 22)				ST Diet (*n* = 18)			
	Baseline	After 4 Weeks	After 8 Weeks	*p*-Value * (Wilcoxon Test)	Baseline	After 4 Weeks	After 8 Weeks	*p*-Value * (Wilcoxon Test)
SBP (mmHg)	117.82 ± 4.57	117.09 ± 3.99	112.82 ± 4.99	<0.001	117.67 ± 10.94	119.33 ± 4.14	112.00 ± 8.84	0.002
SBP-for-age percentile	66.36 ± 19.77	65.00 ± 15.21	50.36 ± 17.21	<0.001	66.78 ± 28.01	75.33 ± 8.43	51.22 ± 20.64	0.002
DBP (mmHg)	73.55 ± 4.19	71.18 ± 6.44	67.09 ± 3.31	<0.001	67.67 ± 6.42	70.56 ± 6.74	65.44 ± 4.60	0.005
DBP-for-age percentile	87.09 ± 9.69	77.64 ± 19.47	66.64 ± 17.10	<0.001	67.22 ± 27.46	75.43 ± 13.73	57.44 ± 19.88	0.010
Heart rate (bpm)	73.27 ± 4.70	73.00 ± 3.46	75.18 ± 4.88	ns	72.44 ± 5.07	76.89 ± 5.75	72.67 ± 3.82	ns
TC (mg/dl)	190.63 ± 19.31	nd	181.05 ± 9.84	0.033	190.54 ± 27.61	nd	173.10 ± 20.52	<0.001
HDL-C (mg/dl)	39.24 ± 10.67	nd	36.87 ± 11.54	0.013	40.98 ± 7.23	nd	40.90 ± 7.23	ns
LDL-C (mg/dl)	110.51 ± 13.62	nd	113.78 ± 12.25	ns	109.79 ± 16.17	nd	114.82 ± 16.06	ns
TG (mg/dl)	216.64 ± 65.41	nd	157.09 ± 61.56	<0.001	206.19 ± 56.00	nd	124.70 ± 40.34	<0.001

SBP—systolic blood pressure; DBP—diastolic blood pressure; TC—total cholesterol; HDL-C—high-density lipoprotein cholesterol; LDL-C—low-density lipoprotein cholesterol; TG—triglyceride; nd—no data (no measurements were taken at this time point); ns—not significant (*p* ≥ 0.05); LGI diet—low-glycemic index diet; ST diet—standard therapy diet; * significant differences between baseline and after 8 weeks from the LGI diet and ST diet groups, respectively.

**Table 7:** Changes in metabolic parameters in LGI and ST diet groups after the 8-week nutritional intervention (mean ± SD).

Variable	LGI Diet (*n* = 22)	ST Diet (*n* = 18)	*p*-Value (Mann–Whitney U Test)
Anthropometrics			
Height (cm)	0.47 ± 0.59	0.58 ± 0.47	ns
Weight (kg)	−4.21 ± 3.48	−3.09 ± 1.35	ns
Weight-for-age percentile	−1.45 ± 1.92	−3.43 ± 3.00	0.027
BMI (kg/m^2^)	−1.48 ± 1.05	−1.26 ± 0.41	ns
BMI-for-age percentile	−2.64 ± 5.47	−3.78 ± 3.14	0.013
Arm circumference (cm)	0.35 ± 1.33	−0.29 ± 0.99	ns
Waist circumference (cm)	−5.20 ± 8.34	−3.64 ± 2.57	ns
Hip circumference (cm)	−2.11 ± 3.98	−3.79 ± 2.49	ns
WHtR	−0.03 ± 0.05	−0.02 ± 0.02	ns
WHR	−0.03 ± 0.07	−0.00 ± 0.03	ns
FM (kg)	−3.20 ± 3.93	−1.87 ± 1.06	ns
FM (%)	−1.59 ± 2.53	−1.22 ± 1.11	ns
FFM (kg)	−1.00 ± 2.18	−1.23 ± 0.83	ns
FFM (%)	1.63 ± 2.49	1.22 ± 1.13	ns
TBW (kg)	−0.61 ± 1.52	−0.53 ± 0.19	ns
TBW (%)	1.38 ± 1.84	1.38 ± 0.69	ns
MM (kg)	−0.74 ± 1.97	−0.66 ± 0.28	ns
MM (%)	1.86 ± 2.41	1.82 ± 0.98	ns
SMM (kg)	−0.43 ± 1.18	−0.38 ± 0.14	ns
SMM (%)	0.96 ± 1.43	0.84 ± 0.78	ns
Cardiometabolics			
SBP (mmHg)	−5.00 ± 3.49	−5.67 ± 4.90	ns
SBP-for-age percentile	−16.00 ± 9.42	−15.56 ± 13.48	ns
DBP (mmHg)	−6.45 ± 5.51	−2.22 ± 2.86	0.008
DBP-for-age percentile	−20.45 ± 19.28	−9.78 ± 12.67	ns
Heart rate (bpm)	1.91 ± 5.17	0.22 ± 2.51	ns
TC (mg/dl)	−9.58 ± 20.52	−17.44 ± 13.23	ns
HDL-C (mg/dl)	−2.36 ± 5.64	−0.08 ± 5.02	0.046
LDL-C (mg/dl)	3.27 ± 17.48	5.03 ± 12.76	ns
TG (mg/dl)	−60.14 ± 32.12	−81.27 ± 29.50	0.030

BMI—body mass index; WHtR—waist-to-height ratio; WHR—waist-to-hip ratio; FM—fat mass; FF—fat-free mass; TBW—total body water; MM—muscle mass; SMM—skeletal muscle mass; SBP—systolic blood pressure; DBP—diastolic blood pressure; TC—total cholesterol; HDL-C—high-density lipoprotein cholesterol; LDL-C—low-density lipoprotein cholesterol; TG—triglyceride; ns—not significant (*p* ≥ 0.05); LGI diet—low-glycemic index diet; ST diet—standard therapy diet.

**Table 8:** Body weight and selected cardiometabolic parameters before and after 8 weeks of nutritional intervention (% of participants).

Variable		Baseline			After 8 Weeks			*p*-Value (Chi-Squared Test)
		Total (*n* = 40)	LGI (*n* = 22)	ST (*n* = 18)	Total (*n* = 40) ^†^	LGI (*n* = 22) ^‡^	ST (*n* = 18) ^§^	
Body weight *	Normal	0	0	0	15	18	11	<0.001 ^†^ <0.001 ^‡^ 0.004 ^§^
	Overweight	40	36	44	40	27	56	
	Obesity	60	64	56	45	55	33	
SBP-for-age percentile	<90	85	82	89	100	100	100	ns ^†^ ns ^‡^ ns ^§^
	≥90	15	18	11	0	0	0	
DBP-for-age percentile	<90	75	55	100	95	91	100	0.015 ^†^ ns ^‡^ ns ^§^
	≥90	25	45	0	5	9	0	
TC	Acceptable	25	27	22	35	18	56	ns ^†^ ns ^‡^ 0.005 ^§^
	Borderline high **	40	27	56	60	82	33	
	High **	35	46	22	5	0	11	
HDL-C	Acceptable	20	18	22	25	27	22	<0.001 ^†^ <0.001 ^‡^ <0.001 ^§^
	Borderline low ***	30	27	34	25	18	34	
	Low ***	50	55	44	50	55	44	
LDL-C	Acceptable	55	64	44	40	45	33	0.008 ^†^ ns ^‡^ <0.001 ^§^
	Borderline high	40	36	44	50	45	56	
	High	5	0	12	10	10	11	
TG	Acceptable	0	0	0	20	18	22	<0.001 ^†^ <0.001 ^‡^ ns ^§^
	Borderline high	10	18	0	20	9	34	
	High	90	82	100	60	73	44	

* Based on the BMI criteria and Polish percentile charts [14] as defined by IOTF [15]: normal for the 5th to 85th percentile, overweight for the 85th to 95th percentile, obesity > 95th percentile; ** the cut points for borderline high and high lipid values represent approximately the 75th and 95th percentiles, respectively [16]; *** low cut points for HDL-C represent approximately the 10th percentile [16]; SBP—systolic blood pressure; DBP—diastolic blood pressure; TC—total cholesterol; HDL-C—high-density lipoprotein cholesterol; LDL-C—low-density lipoprotein cholesterol; TG—triglyceride; ns—not significant (*p* ≥ 0.05); significant differences between baseline and after 8 weeks in the following groups (*p* < 0.05): ^†^ total, ^‡^ LGI diet, ^§^ ST diet; LGI diet—low-glycemic index diet; ST diet—standard therapy diet.

## Data Availability

The datasets used and/or analyzed during the current study are available from the corresponding author upon reasonable request. Currently, we do not want to make the data public because it is part of the research for a planned PhD dissertation.
